# Examining aggressive behavior in patients with epilepsy under treatment with Levetiracetam, Brivaracetam and Perampanel: a comparison to healthy controls

**DOI:** 10.3389/fnbeh.2026.1799427

**Published:** 2026-04-13

**Authors:** Vanessa Murray, Laura Dukek, Salma Lamine, Vesal Moaiyed, Philipp Honrath, Yvonne Weber, Ute Habel, Stefan Wolking, Lisa Wagels

**Affiliations:** 1 Department of Psychiatry, Psychotherapy and Psychosomatics, Faculty of Medicine, RWTH Aachen University, Aachen, Germany; 2 Section Epileptology, Department of Neurology, RWTH Aachen University, Aachen, Germany; 3 Research Center Jülich, Institute of Neuroscience and Medicine: JARA-Institute Brain Structure Function Relationship (INM 10), Jülich, Germany; 4 Center for Computational Life Sciences, RWTH Aachen University, Aachen, Germany

**Keywords:** aggression, antiseizure medication, comorbidities, epilepsy, frustration, revengefulness

## Abstract

**Introduction:**

Epilepsy is frequently associated with behavioral and emotional difficulties. Among antiseizure medications, Levetiracetam, Perampanel, and Brivaracetam are notorious to cause behavioral adverse drug reactions, including increased anger, irritability, and aggression. While these effects are often described in self-report data, experimental studies examining aggressive behavior in patients with epilepsy are scarce. This study aims to assess aggression in patients with epilepsy and compare it to healthy controls, using self-report measures and two experimental provocation paradigms.

**Methods:**

Thirty seven patients with epilepsy treated with Levetiracetam, Perampanel, or Brivaracetam (with an overrepresentation of patients treated with Levetiracetam) and 38 healthy controls completed the Buss and Perry Aggression Questionnaire, Barratt Impulsiveness Scale, Beck’s Depression Inventory, and Affective Style Questionnaire. Aggressive behavior and reaction were investigated using a modified version of the Taylor Aggression Paradigm and the Technical Provocation Paradigm, alongside with emotional state ratings during the paradigms.

**Results:**

Patients with epilepsy reported higher aggression, impulsivity, and depression in the self-report questionnaires compared to healthy controls. However, behaviorally, patients did not show increased aggression. Instead, they showed reduced emotional reactivity to provocation, with lower changes in the emotional state ratings. Higher desire for revenge and higher frustration during the task predicted stronger aggressive responses in the patient group, but not in the control group.

**Discussion:**

Our findings suggest reduced emotional responsiveness to provocation, and a discrepancy between self-perceived and observed aggression in patients with epilepsy. Aggressive responses in this group appeared to be influenced by situational emotions such as revengefulness and frustration. These results underline the need to differentiate between subjective and observable behavioral changes in patients with epilepsy, and of considering the impact of psychiatric comorbidities and emotion regulation difficulties.

## Introduction

Epilepsy is one of the most prevalent neurological disorders, affecting more than 50 million people worldwide. The disease-associated psychosocial burden does not only derive from epilepsy-specific factors, such as seizure frequency and type alone. Psychiatric comorbidities, especially mood and anxiety disorders (Lu et al., 2021), are common in patients with epilepsy (PWE). Treatment with antiseizure medication (ASM) and psychological features like stigma perception and emotion dysregulation can contribute to an impaired quality of life in PWE (Zeber et al., 2007; Srikanth et al., 2021; Tombini et al., 2021; Shen et al., 2022). Previous studies suggest that identifying these contributing factors is key to provide appropriate treatment (Tombini et al., 2021).

Most PWE are treated with ASM with the goal to achieve seizure freedom. However, ASM can also be detrimental to quality of life due to adverse drug reactions (Wang et al., 2022). Whereas many ASMs have unspecific adverse drug reactions in common, like fatigue and cognitive impairment (Carpay et al., 2005), Levetiracetam, Perampanel and Brivaracetam are specifically associated with behavioral adverse drug reactions, including increased aggression and irritability (Hansen et al., 2018; Steinhoff et al., 2021).

Behavioral adverse drug reactions occur significantly more often with Levetiracetam treatment than with other ASMs with incidences ranging from 13.5% to approximately 50% (French et al., 2001; Wieshmann and Baker, 2013). The most frequent effects include irritability, depressive mood, anxiety, and aggression (Weintraub et al., 2007; Chen et al., 2017). Brivaracetam is also associated with the occurrence of adverse drug reactions, such as mood lability and aggressiveness, although with lower frequencies than for Levetiracetam (Hirsch et al., 2018). Some studies have also suggested a potential for Brivaracetam to both improve attention and executive functions and to lower anger levels (Witt et al., 2018; Toledo et al., 2019). Behavioral adverse drug reactions, including anger, irritability, and aggression, have been described in 11–22% of patients treated with Perampanel (Hansen et al., 2018). A dose dependence of adverse drug reactions has been shown, and dose reduction was effective in resolving most of these reactions (Ettinger et al., 2015).

Human aggressive behavior is defined as any behavior with the intention to inflict harm on a target which wants to avoid harm (Baron and Richardson, 1994). Following the revised version of the Frustration-Aggression-Hypothesis, aggression occurs based on prior frustration, particularly if negative affect is high and individual characteristics predispose an individual to an aggressive response (Berkowitz, 1989). At the same time, aggression can be driven by the pleasure and emotional relief of revenge (Chester and DeWall, 2018).

Different approaches to measure aggression have been proposed: interviews, psychometric measures, such as questionnaires, and experimental paradigms. The most utilized (Koch et al., 2025) standardized questionnaire, the Buss and Perry Aggression Questionnaire (BPAQ) (Buss and Perry, 1992), measures trait aggression on four subscales. Experimental tasks use provoking or frustrating events and allow observing and measuring evoked behavioral responses. Modern versions of the Taylor Aggression paradigm (TAP) provoke aggression in competitive social settings (Boccadoro et al., 2021; Koch et al., 2024), whereas the Technical Provocation Paradigm (TPP) (Panagiotidis et al., 2017) uses a non-social setting.

In epilepsy, several case series found increased intensities of aggression, both related and unrelated to the occurrence of epileptic seizures (Kanner, 2004; Sumer et al., 2007; Pandya et al., 2013). Especially studies based on self-report measures such as the BPAQ have shown that PWE report significantly higher levels of aggression than healthy individuals (Shehata and Bateh, 2009; Kato et al., 2011; Seo et al., 2015; Izci et al., 2016). Higher scores of self-perceived aggression negatively correlated with quality of life (Izci et al., 2016) and were associated with medication profiles. Elevated aggression and irritability were reported for Levetiracetam and Perampanel, and Brivaracetam (Brodie et al., 2016; Hansen et al., 2018; Steinhoff et al., 2021). A retrospective analysis of a large database collecting reports of adverse drug reactions among ASMs showed that Perampanel and Levetiracetam had the highest association with aggression (Kawada et al., 2022).

In a placebo-controlled study, adverse drug reactions related to hostility and aggression, psychosis and psychotic disorders, were more common in the Perampanel group than in the placebo group (French et al., 2015). The prescription of Perampanel significantly increased self-reported aggression levels of PWE compared to previously used ASMs (Goji and Kanemoto, 2019). Patients on therapy with Levetiracetam reported higher self-perceived aggression compared to patients treated with other ASMs, and, directly compared to patients who used Perampanel, they showed higher hostility scores (Kawai et al., 2021; Kawai et al., 2022). The evaluation of self-reported adverse drug reactions of patients on therapy with either Levetiracetam or Perampanel showed a more universal negative effect on mood for Levetiracetam. The latter not only increased aggression, but was also associated with depression, restlessness and compulsive behavior. Perampanel in contrast had more specific negative effects on mood, especially increasing the risk for aggression and irritability (von Wrede et al., 2021).

Importantly, these assessments of aggressive traits and behaviors relied on self-reports through questionnaires and only few studies used records from treating physicians (Kanner, 2004; Zhang et al., 2022). To our knowledge, only two animal-based studies using an experimental paradigm to investigate the presence of aggression in epilepsy were published, with one finding no increased aggression (Smolensky et al., 2019) and the other reporting increased aggressive behavior (Suchomelova et al., 2023), and no human studies were found.

Reactive aggression, which evolves as a rather quick and uncalculated response to provocation, has been linked to impulsivity (Vaughan et al., 2023). Increased impulsivity has been reported to occur in some types of epilepsy, whereas other types did not show differences to healthy individuals (Lee et al., 2022b; Gonzalez Stivala et al., 2024) Overall impulsivity is associated with a higher rate of depressive symptoms and tonic-clonic seizures (Lee et al., 2022a) and comorbid psychiatric disorders (Gonzalez Stivala et al., 2024).

Studies on the relationship of impulsivity with either Levetiracetam, Brivaracetam, or Perampanel are sparse. Increased non-planning impulsivity was related to negative adverse drug reactions under therapy with Levetiracetam (Helmstaedter et al., 2008). Higher overall impulsivity in the BIS increased the risk for psychiatric adverse drug reactions on Levetiracetam (Shakeshaft et al., 2021). While Levetiracetam is linked to a broad spectrum of mood changes, including impulsivity, Perampanel is not associated with impulsivity (von Wrede et al., 2021). Specific studies for Brivaracetam were not identified.

This study aimed to assess aggression and impulsivity in PWE compared to healthy controls (HC) by employing two experimental tasks with different contexts in addition to self-report instruments. By combining both behavioral measures and subjective assessments, we intended to compare the results of self-reports and observed aggression responses between patients and HC. We investigated whether self-perceived aggression levels correlate with task behavior. Due to the limited sample size, a comparison between the different ASMs was not intended.

Based on the current state of research on aggression and impulsivity in PWE, and the effects of ASM, we expected that PWE would report higher self-perceived aggression and impulsivity compared to HC (subjective trait aggression hypothesis). We anticipated increased levels of aggressive behavior in the social provocation context (TAP) compared to the control group (social provocation hypothesis), alongside a stronger desire for revenge during the task (motivation hypothesis). We also predicted that PWE would exhibit more aggressive behavior when exposed to frustrating events in the non-social task (TPP) (non-social provocation hypothesis) and report stronger feelings of frustration during this task compared to HC (frustration emotion hypothesis). Furthermore, we assumed that PWE might show discrepancies between their self-perceived aggression and their observed behavioral responses (subjective versus objective aggression hypothesis).

## Materials and methods

### Sample

Inclusion criteria for both participant groups were an age between 18 and 60 (HC) or 70 (PWE), sufficient physical health to participate in the study without any limitations, and the ability to provide written informed consent. Exclusion criteria that were applied to both groups were cognitive impairment, acute severe psychiatric disorders including psychosis or delirium-induced limited consciousness, ongoing substance abuse, insufficient knowledge of German, and contraindications for MRI measurements.

PWE were recruited from both the inpatient ward and the outpatient clinic at the University Hospital RWTH Aachen, Epilepsy Center, Department of Neurology. Additionally, patients who had prior contact to the research team but were treated at external hospitals or neurologists were invited to participate. Additional inclusion criteria for the patient group were a confirmed epilepsy diagnosis and ongoing treatment with one of the following drugs: Levetiracetam, Brivaracetam or Perampanel. Patients with a first-time prescription could participate after a minimum treatment duration of between 1 and 3 weeks depending on the type of medication. Prior to the MRI measurement, the treating physician confirmed the measurement would pose no additional seizure risk to the patient.

HC were recruited from the general population via flyers, posters, and social networks. Additional exclusion criteria for this group were any kind of psychiatric or neurological disorder.

A total of 39 PWE were recruited between February 2023 and July 2024. We recruited 34 patients from the ward and three patients from the outpatient clinic of the Neurology department RWTH Aachen university hospital. Two additional patients who were treated by external neurologists participated in the study as well.

Two patients had to be excluded, one due to withdrawal of the epilepsy diagnosis and one due to severe cognitive impairment, leaving a group of *n* = 37 patients for the analysis. This group consisted of *n* = 22 male patients (59%) and *n* = 15 female patients (41%). The age range was 18–67 years, the mean age was 32.73 years (*SD* = 13.31).

38 HC were recruited and included between September 2023 and September 2024. The HC group consisted of *n* = 16 male participants (42%) and *n* = 22 female participants (58%). The age range in this group was 18–57 years, with a mean age of 27.76 years (*SD* = 12.82).

#### Taylor aggression paradigm

**FIGURE 1:**
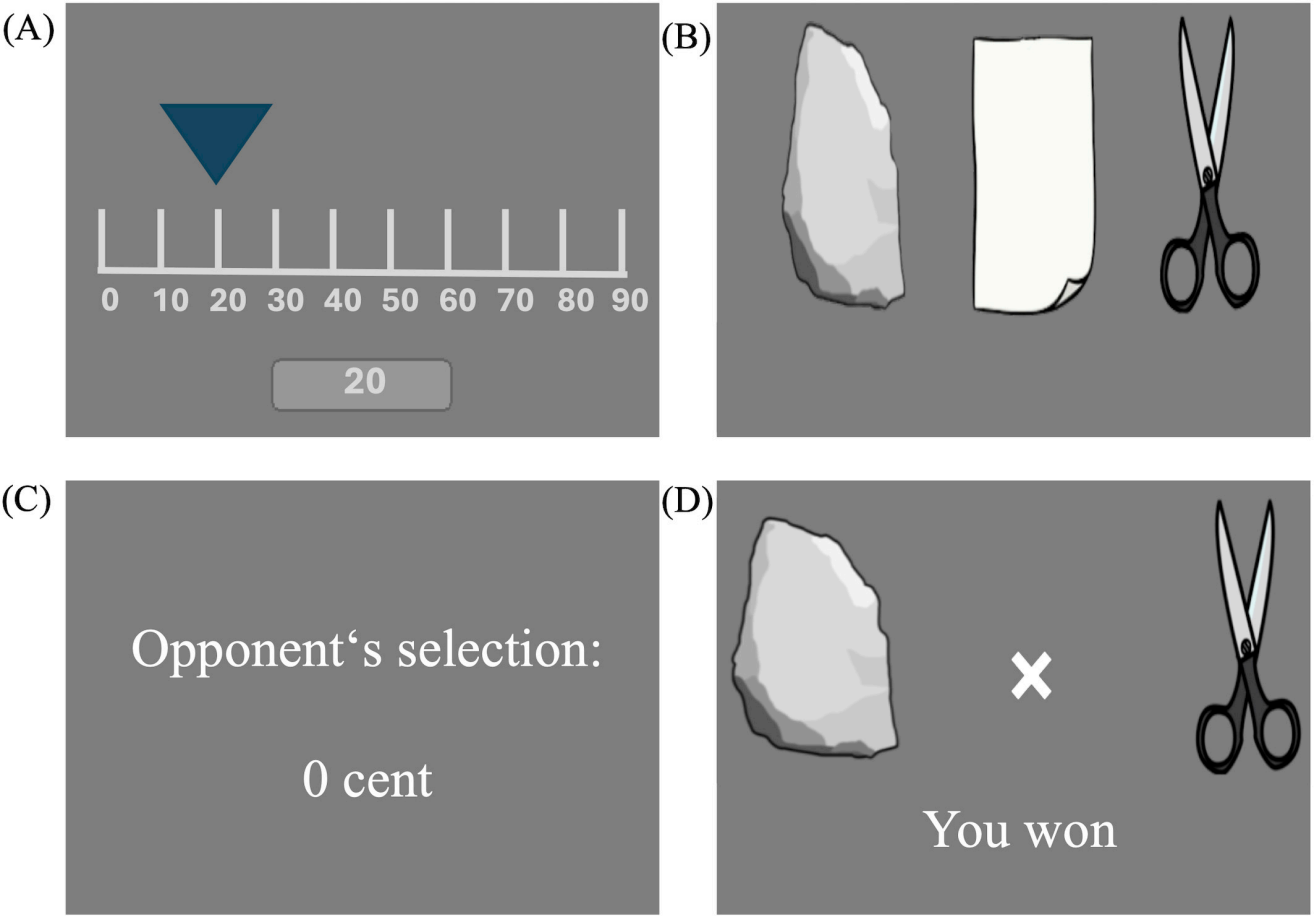
Steps of a TAP trial as displayed on the screen in the scanner: **(A)** Selection of money deduction, **(B)** selection of rock, paper, or scissors, **(C)** display of the opponent’s money selection, **(D)** result of the trial.

A modified MRI compatible version of the TAP was programmed and carried out via PsychoPy version 2021.1.2. In the paradigm participants could select their answers by pushing a button. The button box system was placed in the participants’ right hand before starting the task. Before each trial, the participants selected a monetary deduction between 0 and 100 cents as a punishment to impose on the opponent in case they won the upcoming trial. After the choice of the rock, paper, or scissors, participants first received feedback on how much money the opponent chose and then on who won the trial (Figure 1).

To enhance authenticity, a cover story about a real opponent was implemented. The experiment leader referenced the opponent in the instruction videos and simulated phone calls to contact the person responsible for the ostensible opponent who would perform the task as well. While the participant was in the scanner and waiting for the task to start, the experiment leader would talk to the participant through the speakers of the MRI and again pretend to reach out to opponent‘s team and make sure both teams are ready.

Participants completed the game in three blocks with 20 trials each. The three blocks had different levels of provocation, established through different ranges of predefined money deduction by the ostensible opponent. Low provocation was defined as money deduction between 0 and 40 cents, medium provocation as deduction between 40 and 70 cents, and high provocation as deduction between 70 and 100 cents. The order in which the blocks appeared was either: low, high, medium in version “A”; or it was: medium, low, high in version “B.” PWE were randomized to either perform version A or version B, whereas HC only performed version A.

After each block, the participants answered questions about happiness, satisfaction, sadness, anger, their own fairness and desire for revenge on a 5-point Likert scale (low to high), and about their perceived opponent’s fairness in categorical responses, being “fair in most trials,” “unfair in most trials,” or “fifty-fifty.”

#### Technical provocation paradigm

An MRI compatible version of the TPP (Panagiotidis et al., 2017) was programmed and carried out using presentation version 22.1 build 01.21.21.

Participants were instructed to precisely drop as many balls as possible into a target container with a narrow opening at the top. Participants saw a screen displaying a target container, a ball, and a plate for coin collection. Before starting the task, a joystick was placed in the participants’ right hand, and a button box was placed in their left hand. The joystick was calibrated after placement and before the first use. The experiment leader then verified that the participant could hear a sound that would also appear during the task.

**FIGURE 2:**
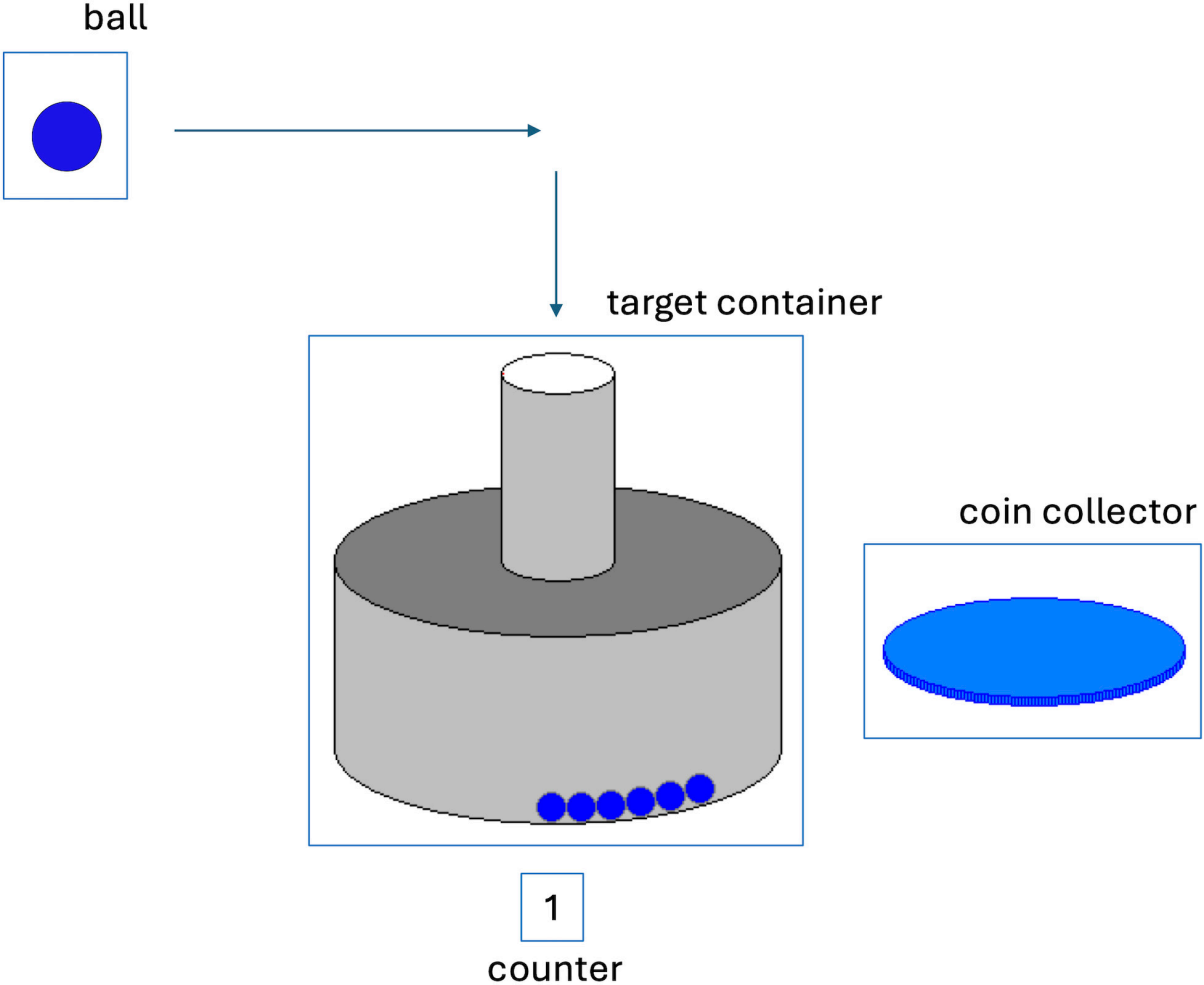
Depiction of the TPP setting with a ball moving horizontally from left to right, and an open container in which the ball should be dropped. The blue balls in the target container show previous successful trials. The counter shows that the last trial was a successful trial. The coin collector would show coins that are earned for two successful trials in a row.

In each trial, a ball automatically moved across the screen horizontally from the top left corner to the top right corner of the screen. By pulling on the joystick at the right time, the ball would drop vertically down into the target container (Figure 2). A successful trial was followed by a sound representing a dropping coin. A failed trial was followed by a sound representing a crashing bomb. For each two successful trials in a row, the participants were rewarded with a coin appearing on the coin-collection plate.

This paradigm included four types of provocation errors. The two existing versions of the paradigm each included two different ones, to simulate either visual or mechanical disruptions. One version confronted the participants with a malfunction of the joystick. In seven predefined trials, the ball failed to drop down despite pulling the joystick, displaying the feedback “Please move the joystick.” In seven other trials, the ball dropped down too early without pulling the joystick, combined with the feedback “Way too early.” The other version of the paradigm prevented the participants from scoring by flashing a black screen in seven trials and a loading icon in seven other trials, always right in the critical moment when the ball passed the target container.

The paradigm consisted of 80 trials organized into eight equally long blocks. Blocks three, five, six and eight included a total of 14 manipulated trials. After each block, the participants answered nine questions including satisfaction, happiness, sadness, anger, frustration, motivation, arousal, valence, and perceived control. Each item was answered on a Likert scale ranging from one (lowest) to five (highest).

#### Questionnaires and structured interview

We assessed depressive symptoms using the German translation (Hautzinger et al., 2006) of the second version of the Beck‘s Depression Inventory (BDI) (Beck et al., 1996). The German translation (Herzberg, 2003) of the BPAQ (Buss and Perry, 1992) was employed to investigate aggression, and the German translation (Graser et al., 2012) of the Affective Style Questionnaire (ASQ) (Hofmann and Kashdan, 2010) was employed to investigate emotion regulation strategies.

To assess impulsivity, study 1 (PWE) used the German version (Preuss et al., 2008) of the BIS-11 (Patton et al., 1995). Study 2 (HC) used the German version (Meule et al., 2011) of the BIS-15 (Spinella, 2007). Only overlapping items of both questionnaires were used for the analysis (representing the BIS-15).

German versions (Beesdo-Baum et al., 2019b,a) of the structured clinical interview for DSM-5 personality disorders (SCID-5-PD) (First et al., 2016b) and the structured clinical interview for DSM-5 clinical version (SCID-5-CV) (First et al., 2016a) were applied to assess diagnostic criteria for a psychiatric disorder according to the DSM-5.

### Procedure

Eligible PWE who met all the inclusion criteria and gave their written informed consent were scheduled for the fMRI measurement, the completion of the four self-report questionnaires and the structured clinical interview. For inpatients, the MRI session was scheduled for the last day of their hospital stay to allow continuous video-EEG-monitoring before the measurement. The questionnaires and the SCID-interviews were done either before or after the measurement, sometimes on a different day based on the patients’ availability. Before the patients went into the 3 Tesla Prisma Scanner (Siemens) placed at the University Hospital RWTH Aachen, they viewed instructional videos explaining the in-scanner tasks and introducing that they would play against an opponent. Any open questions regarding the tasks were answered by the experiment leader, before starting the MRI measurement. The MRI data were acquired in the following order: resting state fMRI, task-based fMRI (TPP and TAP), T1 weighted MPRAGE anatomical scan.

After completion of the appointments, the patients were debriefed by the experiment leader. The study aims and any simulated aspects of the experiments were clarified, including the ostensible opponent and manipulated trials. Any remaining questions were answered before ending the study participation by handing out the financial compensation.

In this study, we only analyzed the behavioral data collected during the paradigms. The fMRI data is part of a separate study (Moaiyed et al., 2025).

HC were invited to participate in a single-day protocol with mostly parallel MRI session and self-report questionnaires after the MRI session. In addition to the protocol used in the patient group, HC completed additional self-report questionnaires, they performed an additional in scanner task, the voodoo doll task (DeWall et al., 2013) (which patients answered in a paper-pencil questionnaire), provided breath and saliva samples between the tasks, and they were asked to participate in an online follow-up survey.

Different to the HC group, PWE repeated all measures except for the SCID in a second appointment after a minimum time period of 4 weeks. These items and additional data were not considered for the analysis in this paper and will not be further addressed.

### Preprocessing of the behavioral data

For the analysis of the TAP, aggression was operationalized as the amount of money participants chose to deduct from their opponent in each trial. Means were calculated for the three different provocation conditions: low, medium, and high. Subjective ratings after each block indicated the condition-related affective state. Revengefulness was selected as the primary affective state, emerging from the competitive interpersonal setting and as a retaliatory motive.

For the analysis of the TPP, aggression was operationalized as the intensity of joystick use. The area under the curve (AUC), representing how strong the joystick was pulled, was calculated for each of the 80 trials. Two means were then calculated for each participant, a mean AUC in neutral conditions (blocks without provocation trials), and for provocation conditions (blocks containing provocation trials). Ratings after each of the eight blocks were then calculated as mean ratings after the provocation condition, and as mean ratings after the neutral condition. We focused on frustration, arising from the performance-related errors in this paradigm.

The number of actual failed trials the participants scored in the two conditions operationalized successfulness in the task. For each participant, two means were calculated. A mean number of failed trials was calculated for neutral blocks and for provocation blocks. For the provocation blocks, only self-caused fails were considered, and fails due to preprogrammed errors were excluded, to focus specifically on the participant’s real task performance.

### Data analysis

The collected data was analyzed using IBM SPSS Statistics (version 19.0.2.0) and R Studio (version 2024.04.1+748). Graphs and Figures were created in R Studio and Microsoft Excel [version 16.89.1 (24091630)]. Given that the sample size exceeded 30 participants in both groups, the Central Limit Theorem (CLT) was applied to assume a normal distribution (Kwak and Kim, 2017).

Missing data in single questionnaire items was imputed using the median imputation method. Outlier detection was performed using visual methods (boxplots), and mild outliers were kept in the dataset. Two identified extreme outliers within two variables were retained as they were not due to data entry errors but depicted the variability within the groups.

Levene’s test for homogeneity of variance was performed, and if variances were equal, independent samples *t*-tests were used for group comparisons of continuous variables. If variances were unequal, Welch’s *t*-test was used. Nominal variables were compared between groups by conducting a chi-square test of independence.

Effect sizes were measured and reported by Cohen’s *d*, and they were interpreted as follows: 0.2 = small, 0.5 = medium, 0.8 = large (Cohen, 1988).

To examine the effects of the study group and the task provocation conditions on affective state ratings (revengefulness in the TAP, frustration in the TPP), we used linear mixed effects models using the lme4 package in R (Bates et al., 2015).

A linear mixed effects model was estimated to predict aggressive behavior operationalized as average money deductions in the TAP, again using the lme4 package (Bates et al., 2015). This model included the fixed factors study group (between-subject factor levels: PWE, HC), TAP condition (within-subject factor levels: low, medium, high provocation), gender (between-subject factor levels: male, female), and TAP version (between-subject factor levels: version A, version B). Revengefulness ratings during the task were included as continuous factor in a three-way interaction with the TAP condition and the study group. A random intercept for participants was included to account for variability within individuals in baseline aggression and repeated measures across TAP trials. The final model was:

modelATEP < − lmer(Money∼ HP_Group +TAP_ condition + TAP_Version + Gender + TAP_condition:TAP_ Revengefulness:HP_Group + (1|Study_ID), na.action = “na.omit,” data = ATEP).

A similar mixed-effects model was tested to predict joystick AUCs in the TPP. The model included the fixed factors study group (between-subject factor levels: patients, HC), TPP block (within-subject factor levels: neutral, provocation), gender (between-subject factor levels: male, female), and a three-way interaction between frustration ratings, the study group, and the TPP condition. A random intercept for participants was included to account for repeated measures. The final model was:

modelATEP_TPP_AUC < − lmer(AUC_block∼ HP_Group + TPP_block + Gender + TPP_Frustration:TPP_block:HP_Group + (1|SNumber), na.action = “na.omit,” data = ATEP_TPP).

The level of statistical significance was set at *p* < 0.05. Satterthwaite’s approximation was applied to assess the significance of fixed effects in the models for TAP and TPP. Estimated marginal means were computed to investigate significant effects and pairwise comparisons were conducted as *post-hoc* tests for the models. Bonferroni correction was applied to adjust for multiple comparisons.

Moderation was tested using the lm() function for linear regression (R Core Team, 2024), with frustration ratings as depended variable and the mean number of failed trials, the study group, and their interaction as predictors.

### Ethics and study registration

The two studies included for data collection were registered in the German Clinical Trials Register (study including the HC population: DRKS00032177, study including the patient population: DRKS00030739). They were approved by the ethics committee of the medical faculty of RWTH Aachen University under the numbers CTC-A-23-049 (HC) and CTC-A-20-210 (PWE). Both studies align with the principles of the Declaration of Helsinki and the guidelines for Good Clinical Practice.

## Results

### Sample characteristics

Three patients did not perform or not complete the TPP due to technical problems, resulting in a sample of *n* = 34 for all analysis involving the TPP data. The TAP was not performed by six patients and not completed by two additional patients, due to either technical problems or discomfort during the fMRI measurement, resulting in *n* = 29 patients for the analysis of the TAP data. Two patients did not fill a complete questionnaire (1 × ASQ, 1 × BPAQ) and were therefore not considered for the analysis involving the respective questionnaire. Furthermore, we detected one missing answer in three data sets and two missing answers in one data set. Answers were missing randomly, and we therefore chose median imputation at item level to impute the data sets.

All 38 participants in the HC group completed the TAP and all questionnaires. Four healthy participants had several missing data in the TPP due to technical problems, resulting in *n* = 34 for analysis involving the TPP.

In the patient group, 22 patients (59.5%) were male, and 15 patients (40.5%) were female, while in the HC group, 16 participants (42.1%) were male, and 22 participants (57.9%) were female. Gender distribution was not statistically significantly different, χ^2^(1, *N* = 75) = 2.26, *p* = 0.133, Φ = 0.17. The mean age in both groups was similar, with PWE being *M* = 32.73 years (*SD* = 13.31) on average and HC being *M* = 27.76 years (*SD* = 12.82) on average, *t*(73) = 1.64, *p* = 0.104, *d* = 0.38. For a detailed description of clinical data of the PWE group, including e.g., the type of epilepsy and drug dosages (see Table 1).

**TABLE 1:** Demographic and clinical data of the PWE group.

Variable	Value
Total number of patients, *n*	37
Sex, female, *n* (%)	15 (40.5)
Age, years, mean ± SD	32.37 ± 13.31
Age at epilepsy onset, years, mean ± SD	22.86 ± 15.27
Duration of epilepsy, years, mean ± SD	10.03 ± 12.34
Epilepsy diagnosis, n (%)	
FE	21 (56.7)
GGE	5 (13.5)
Other	3 (8.1)
Unknown	8 (21.6)
MRI lesion, n (%)	
Yes	16 (43.2)
No	20 (54.1)
Unknown	1 (2.7)
Target ASM, n (%)	
LEV	26 (70.3)
BRV	6 (16.2)
PER	4 (10.8)
BRV + PER	1 (2.7)
Daily ASM dosages, mg, mean ± SD (range)	
LEV	1,780 ± 947.36 (1,000–4,000)
BRV	150.00 ± 50.00 (100–200)
PER	5.20 ± 1.10 (2–6)
Concomitant ASM, n (%)	
LTG	13 (35.1)
LCM	11 (29.7)
TPM	4 (10.8)
CNB	2 (5.4)
VPA	2 (5.4)
Psychiatric comorbidities, n (%) *	
Depression (F32.-/F33.-)	12 (32.4)
Post-traumatic stress disorder (F43.1)	7 (18.9)
Personality disorder (F60.0 -, F21)	4 (10.8)
Anxiety disorders (F40. 0-, F41.0-)	2 (5.4)
Harmful substance use (F10. 0-, F12.0-)	2 (5.4)
ADHD (F90.0)	1 (2.7)
None	20 (54.1)

SD, Standard deviation; FE, Focal Epilepsy; GGE, Genetic Generalized Epilepsy; LEV, Levetiracetam; PER, Perampanel; BRV, Brivaracetam; LTG, Lamotrigine; LCM, Lacosamide; TPM, Topiramate; CNB, Cenobamate; VPA, Valproate; ADHD, Attention Deficit/Hyperactivity Disorder.

* Present and past diagnoses based on the SCID, *n* = 9 (24.3%) were diagnosed with one psychiatric disorder, *n* = 6 patients (16.2%) had two different diagnoses, and *n* = 2 patients (5.4%) had three different diagnoses. 20 patients met no diagnostic criteria.

### Questionnaires

Mean values and statistics for group comparisons of all questionnaire data are depicted in Table 2. When comparing the average BPAQ scores for trait aggression between PWE and HC, we observed a trend for higher aggressiveness in patients. In addition to the total score, the subscale of hostility and the subscale of physical aggression showed the same tendency, with a medium effect size. The groups did not significantly differ on the subscales of verbal aggression and affective aggression.

**TABLE 2:** Group comparisons with mean and standard deviation of self-reports on aggression, impulsivity, depressive symptoms and emotions regulation strategies.

Questionnaire	PWE		HC		*t*	*p*	Cohen’s
	*M*	*SD*	*M*	*SD*			*d*
BPAQ total	61.83	12.15	56.47	11.10	1.98	0.051	0.46
BPAQ physical aggression	16.00	4.61	14.03	4.26	1.91	0.060	0.45
BPAQ verbal aggression	13.06	2.71	12.50	2.73	0.88	0.383	0.20
BPAQ hostility	18.58	5.21	16.34	4.53	1.98	0.052	0.46
BPAQ anger	14.19	4.37	13.60	4.52	0.57	0.571	0.13
BIS total	30.38	5.18	27.82	5.27	2.13	0.037	0.49
BIS attention	9.35	2.37	9.16	2.20	0.37	0.715	0.09
BIS motor	10.11	2.32	9.61	2.25	0.95	0.344	0.22
BIS non-planning*	10.92	2.24	9.05	2.92	3.10	0.003	0.72
BDI total*	8.84	8.65	3.79	4.47	3.16	0.003	0.74
ASQ total	67.28	7.78	67.00	9.09	0.14	0.888	0.03
ASQ conceal	3.14	0.55	2.89	0.74	1.60	0.114	0.37
ASQ adjust	3.49	0.60	3.61	0.67	−0.78	0.436	−0.18
ASQ tolerate	3.59	0.59	3.82	0.68	−1.50	0.138	−0.35

M, mean; SD, standard deviation; *p*-values are unadjusted, Bonferroni corrected significance level is α* = 0.0036.

Patients rated themselves significantly higher on the non-planning impulsivity subscale of the BIS. The difference in the total score was not significant, although a medium sized effect was observed here. Scores on the attentional impulsivity subscale, and the motor impulsivity subscale were not significantly higher in the patient group. Emotion regulation scores assessed in the ASQ showed no significant differences between patients and HC in the total score. Subscale scores did not show significant differences either. The rating of depressive symptoms in the BDI was significantly higher in PWE than in healthy participants. For an overview on the BDI cut-offs for depression and the SCID-CV diagnoses (see Table 3). A moderate to strong positive Pearsons’s correlation was found between the BDI total score and a current depression diagnosis in the SCID, *r* = 0.49, *p* = 0.003. In the HC group, two participants indicated mild to moderate symptoms of depression, yet participants did not meet the diagnostic criteria for a depressive episode.

**TABLE 3:** Frequencies and percentages at different cut-offs for depression severity based on BDI and SCID-CV diagnoses.

Depression severity	BDI–PWE	SCID-CV–PWE	BDI–HC
No depression	25 (67.6%)	33 (89.2%)	36 (94.8%)
Mild depression	8 (21.6%)	1 (2.7%)	1 (2.6%)
Moderate depression	2 (5.4%)	2 (5.4%)	1 (2.6%)
Severe depression	2 (5.4%)	1 (2.7%)	0

As an additional control accounting for the influence of a SCID-CV/-PD diagnosis on questionnaire results, *post-hoc*, a series of ANCOVAs were conducted for questionnaire scores that showed significant group differences at the uncorrected level. Results revealed that most group differences did not remain significant after adjusting for the presence of a psychiatric comorbidity, and that the SCID diagnosis was a significant covariate for BPAQ total scores and BDI scores. Detailed results can be found in Table 4.

**TABLE 4:** Statistical effects of the ANCOVA with group (PWE vs. HC) and SKID diagnosis as covariate on self-reports on aggression, impulsivity and depression.

Dependent variable	*F*(1, 72) group	*P* group	Partial η^2^ group	*F*(1, 72) covariate	*P* covariate	Partial η^2^ covariate	*R*^2^ model
BPAQ total	0.18	0.672	0.00	6.52	0.013	0.08	0.13
BPAQ hostility	1.86	0.177	0.03	0.42	0.519	0.01	0.06
BIS total	0.67	0.417	0.01	3.76	0.056	0.05	0.11
BIS non planning	6.73	0.012	0.09	< 0.01	0.970	< 0.01	0.12
BDI total	2.29	0.134	0.03	5.90	0.018	0.08	0.189

*p*-values are unadjusted, Bonferroni corrected significance level is α = 0.01.

A multiple linear regression (Table 5) was calculated to examine whether the variables study group (PWE, HC), age, gender, the BDI total score, the BIS total score, or the ASQ total score and the interactions of each variable with the group could predict self-reported aggression (BPAQ total score). The overall model was statistically significant, *F*(11, 65) = 4.25, *p* < 0.001, *R*^2^ = 0.42, adjusted *R*^2^ = 0.32. Yet, no individual predictor and no interaction reached statistical significance.

**TABLE 5:** Statistical effects, regression coefficient, standard error of the multiple linear regression predicting trait aggression.

Predictor	*B*	SE	*t*	*P*
(Intercept)	57.08	2.79	20.44	< 0.001
HP Group	−4.08	4.06	−1.01	0.318
Gender	3.52	3.73	0.95	0.348
Age	0.02	0.14	0.17	0.864
BDI	0.50	0.40	1.24	0.221
BIS	0.61	0.32	1.90	0.062
ASQ	−0.32	0.19	−1.65	0.104
Group × gender	8.14	5.39	1.51	0.136
Group × age	0.36	0.20	1.85	0.070
Group × BDI	0.08	0.46	0.18	0.860
Group × BIS	−0.47	0.49	−0.95	0.346
Group × ASQ	0.13	0.33	0.39	0.699

SE, standard error.

### Taylor aggression paradigm

#### Revengefulness levels

**FIGURE 3:**
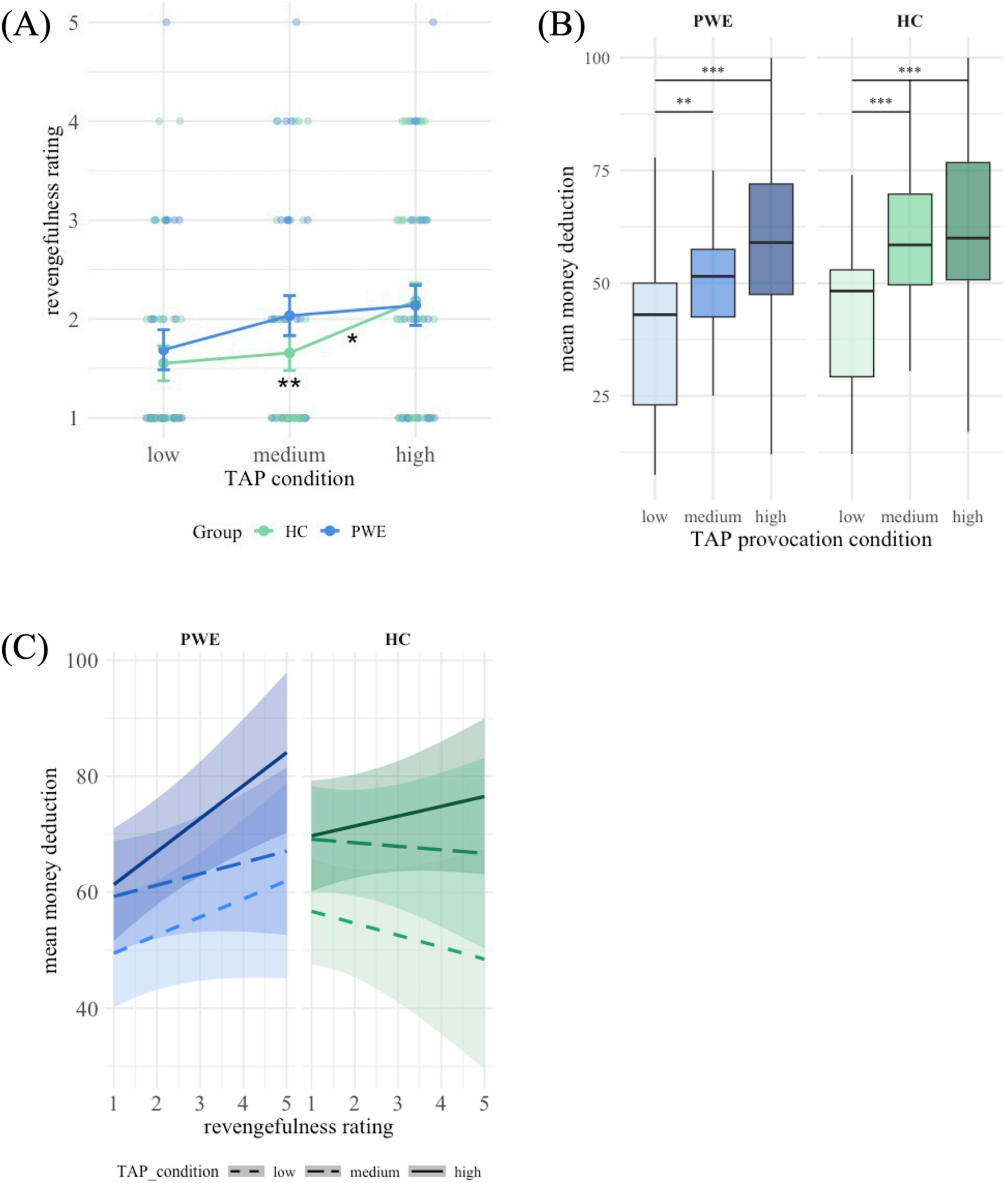
**(A)** Mean scores and standard error of the mean for revengefulness across TAP conditions, *p < 0.05 for difference between medium and high provocation in the HC group, ** *p* < 0.01 for difference between low and high provocation in the HC group. **(B)** Box plots for mean money deduction across TAP conditions. **(C)** Interaction of revengefulness ratings and mean money deduction across TAP conditions. *p < 0.05, **p < 0.01, ***p < 0.001.

Investigating revengefulness ratings during the TAP conditions (Figure 3A) showed a significant main effect of the TAP condition on revengefulness ratings, *F*(2, 130) = 6.59, *p* = 0.002. Bonferroni corrected *post-hoc* comparisons showed that revengefulness ratings were significantly lower in the low provocation compared to the high provocation condition, *t*(130) = 3.61, *p* = 0.001. The difference between low and medium provocation, *t*(130) = −1.51, *p* = 0.403, and the difference between medium and high provocation, *t*(130) = 0.32, *p* = 0.111, were not statistically significant. Comparing estimated marginal means within the two groups revealed that statistical differences in revengefulness ratings were only found within the HC group, not in the PWE group (Table 6). The study group (PWE and HC) had no significant effect on revengefulness ratings, *F*(1, 65) = 0.57, *p* = 0.453, neither had the interactions of the group and the TAP condition, *F*(2, 130) = 1.01, *p* = 0.368.

**TABLE 6:** Statistical effects, estimate and standard error for within group comparisons of revengefulness ratings across TAP provocation conditions.

Contrasts	Estimate	*SE*	*t*(130)	*P*
HC				
Low vs. medium	−0.11	0.20	−0.54	1.000
Medium vs. high	−0.53	0.20	−2.68	0.025
Low vs. high	−0.53	0.20	−3.21	0.005
PWE				
Low vs. medium	−0.35	0.23	−1.53	0.384
Medium vs. high	−0.10	0.23	−0.46	1.000
Low vs. high	−0.45	0.23	−1.99	0.145

*p*-values are Bonferroni corrected.

#### Revengefulness related aggression effects in the TAP

In the linear mixed-effects model, a significant main effect of the TAP condition was observed, *F*(2, 132.15) = 4.33, *p* = 0.015, showing that participants’ aggressive responses changed across the three different provocation levels (Figure 3B). *Post-hoc* estimated marginal means of money deductions were the lowest in the low provocation condition (*M* = 42.1, *SE* = 3.00), higher in the medium provocation condition (*M* = 53.3, *SE* = 2.98), and highest in the high provocation condition (*M* = 57.3, *SE* = 3.00). Bonferroni-corrected pairwise comparisons indicated that the difference between the low and medium provocation, *t*(127) = −5.81, *p* < 0.001, as well as the difference between the low and high provocation, *t*(130) = −7.56, *p* < 0.001, were statistically significant. The difference between the medium and high provocation did not reach statistical significance, *t*(128) = −2.01, *p* = 0.139.

The effect of the study group, PWE vs. HC, showed a trend toward significance, *F*(1, 102.94) = 3.05, *p* = 0.084. Estimated marginal means showed that overall money deductions of patients (*M* = 48.4, *SE* = 3.51) were lower than those of HC (*M* = 53.5, *SE* = 4.61).

There was no significant effect of the TAP version (different orders of the provocation conditions) on money deductions, *F*(1, 62.97) = 0.60, *p* = 0.440. However, a significant effect of gender was observed, *F*(1, 63.18) = 4.12, *p* = 0.047. Comparisons of estimated marginal means revealed that males (*M* = 55.6, *SE* = 3.55) deducted higher amounts of money than females (*M* = 46.2, *SE* = 3.67).

A trend-level three-way interaction was observed between group, provocation condition, and revengefulness ratings during the task, *F*(6, 140.07) = 2.06, *p* = 0.062. Estimated simple slopes of revengefulness predicting aggression across the provocation conditions showed that among PWE, higher revengefulness significantly predicted higher money deduction in the high provocation condition (Table 7). Revengefulness ratings had no significant associations with money selections in the low or medium provocation block in the patient group. In the HC group, this association was not significant in any condition. The interaction slopes are visualized in Figure 3C.

**TABLE 7:** Simple effect slopes of revengefulness predicting aggression separately for TAP provocation conditions and groups.

Contrasts	β	*SE*	df	*t*	*P*
HC					
Low	−2.06	2.40	163	−0.86	0.392
Medium	−0.61	2.06	161	−0.30	0.768
High	1.70	1.72	159	0.99	0.326
PWE					
Low	3.12	2.05	142	1.52	0.130
Medium	1.95	1.81	144	1.08	0.283
High	5.70	1.75	142	3.26	0.001

SE, standard error.

Comparing the estimated slopes of the revengefulness ratings predicting money deductions within groups (Table 8) revealed no significant differences across the provocation conditions in the HC group. The difference between low and high provocation showed a trend level significance, which did not remain significant after Bonferroni correction. In the patient group, the association differed marginally between high and medium provocation, which again was not significant at the corrected level.

**TABLE 8:** Pairwise within group comparisons of simple slopes for revengefulness predicting aggression contrasting TAP conditions.

Contrasts	β	*SE*	df	*t*	*P*
HC					
Low vs. medium	−1.45	2.33	132	−0.62	0.534
Low vs. high	−3.76	2.27	136	−1.66	0.100
Medium vs. high	−2.30	2.07	134	−1.11	0.268
PWE					
Low vs. medium	1.18	2.17	132	0.85	0.589
Low vs. high	−2.57	2.27	135	0.50	0.259
Medium vs. high	−3.75	2.00	132	0.15	0.063

SE, standard error; *p*-values are unadjusted, Bonferroni corrected significance level is α = 0.0167.

#### Controlling for the influence of psychiatric comorbidities

As *post-hoc* analysis, an alternative linear mixed model was tested including the presence of a SCID-based diagnosis as covariate to control for potential influence. Otherwise, this model included the same fixed effects and interactions as the previously presented model. Comparing the two models showed that the inclusion of the SCID diagnosis did not significantly improve the model fit, χ^2^(1) = 1.41, *p* = 0.235. The reported effects of the previous model remained significant in the model controlling for psychiatric comorbidities, except for the trend level effect of the study group (for detailed results see Supplementary material).

### Technical provocation paradigm

#### Frustration levels

**FIGURE 4:**
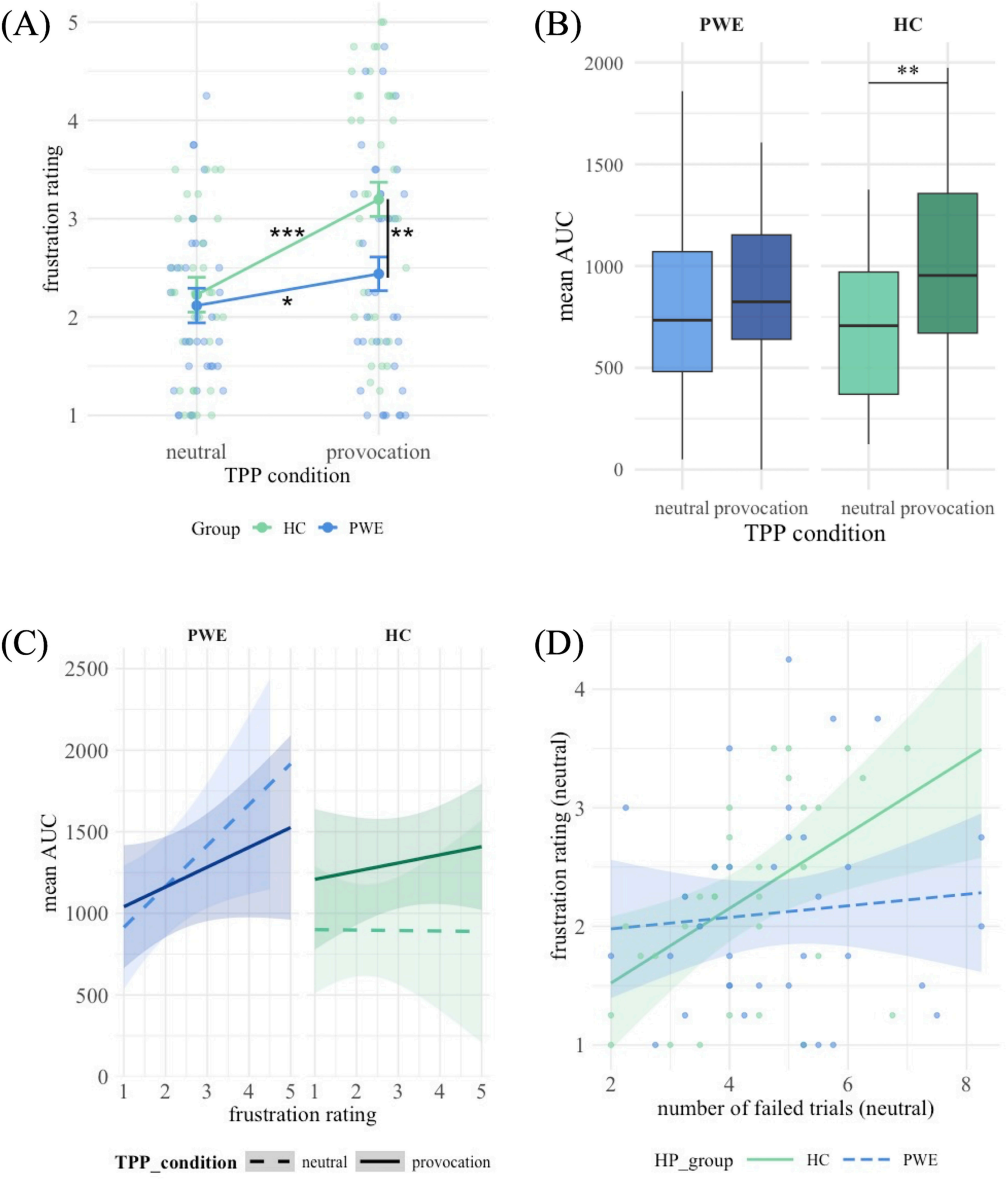
**(A)** Mean scores and standard error of the mean for frustration across TPP conditions. **(B)** Box plots for mean AUCs across TPP conditions. **(C)** Interaction of frustration ratings and mean AUCs across TPP conditions. **(D)** Moderation of the association between failed trials and frustration in the TPP by the group. *p < 0.05, **p < 0.01, ***p < 0.001.

Investigating frustration ratings during the TPP conditions (Figure 4A), a significant main effect of the TPP condition on frustration ratings was observed, *F*(1, 66.32) = 36.09, *p* = < 0.001. *Post-hoc* comparisons showed that frustration ratings were significantly lower in the neutral compared to the provocation condition, *t*(70.9) = 1.95, *p* < 0.001. The study group (PWE and HC) showed a trend level effect on frustration ratings, *F*(1, 69.62) = 3.82, *p* = 0.055. HC rated themselves significantly more frustrated than PWE in the provocation condition, the difference in the neutral condition was not significant (Table 9). There was a significant interaction between the study group and the TPP condition, *F*(1, 66.32) = 9.04, *p* = 0.004. In both groups, the difference in frustration ratings after the provocation condition blocks compared to the neutral condition blocks were statistically significant, though the difference was stronger in the HC group (Table 9).

**TABLE 9:** Contrasts of pairwise within and between group comparisons of frustration ratings.

Contrasts	Estimate	*SE*	*t*	*P*
Within group contrasts				
HC: Neutral vs. provocation	−0.97	0.15	−6.36	< 0.001
PWE: Neutral vs. provocation	−0.32	0.15	−2.112	0.038
Between group contrasts				
Neutral: HC vs. PWE	0.11	0.25	0.44	0.66
Provocation: HC vs. PWE	0.76	0.24	3.10	0.003

SE, standard error; *p*-values are Bonferroni corrected.

#### Frustration related aggression effects in the TPP

The analysis of the linear mixed-effects model revealed no significant effect of the TPP block, *F*(1, 66.82) = 1.56, *p* = 0.215. The main effect of the study group was also not statistically significant, *F*(1, 128.28) = 0.50, *p* = 0.483 (Figure 4B). There was a significant effect of gender, *F*(1, 65.61) = 6.47, *p* = 0.013. *Post-hoc* estimated marginal means showed that the AUC of males (*M* = 766, *SE* = 109) was lower than the AUC of females (*M* = 1,174, *SE* = 118).

Additionally, there was a significant three-way interaction (Figure 4C) between the study group, the TPP condition blocks, and average frustration ratings, *F*(1, 89.67) = 3.20, *p* = 0.017. In the patient group, frustration was a significant positive predictor of the AUC in neutral conditions (Table 10). Comparing the slopes between the two conditions showed that neither in the patient group, *t*(73.8) = 1.43, *p* = 0.158, nor in the HC group, *t*(77.7) = −0.61, *p* = 0.541, there was a significant difference.

**TABLE 10:** Simple slope effects of frustration ratings predicting aggression separate for TPP condition and group.

Condition	β	*SE*	df	*t*	*P*
HC					
Neutral	−2.84	114.0	122	−0.03	0.980
Provocation	50.03	77.1	127	0.65	0.518
PWE					
Neutral	50.53	115.0	131	2.17	0.031
Provocation	121.37	89.3	132	1.36	0.176

SE, standard error.

#### Controlling for the influence of psychiatric comorbidities

To investigate whether a clinical psychiatric diagnosis would explain differences in the AUCs, another model was calculated with the presence of a SCID diagnosis as a covariate. Compared to the original model, no significant improvement in model fit by adding the SCID diagnosis was found, χ^2^(1) = 0.08, *p* = 0.776. Effects remained the same as in the original model (for detailed results see Supplementary material).

#### The effect of task performance

Two moderation analyses examined whether the number of self-caused failed trials predicted frustration ratings differently for HC and PWE (for detailed results see Table 11).

**TABLE 11:** Statistical effects of regression-based moderation analysis predicting frustration ratings in the neutral and provocation TPP condition.

Predictor	Condition	Estimate	*SE*	*t*	*P*
Fail trials	Neutral	0.32	0.11	2.99	0.005**
Group (PWE vs. HC)	Neutral	0.99	0.66	1.49	0.142
Fail trials × group interaction	Neutral	−0.27	0.14	−1.89	0.064
Fail trials	Provocation	0.21	0.23	0.88	0.382
Group (PWE vs. HC)	Provocation	0.43	0.98	0.44	0.659
Fail trials × group interaction	Provocation	−0.35	0.27	−1.28	0.204

** = p <0.01, SE, standard error. Neutral model: *F*(3, 64) = 3.01, *p* = 0.037, adjusted *R*^2^ = 0.08. Provocation model: *F*(3, 64) = 2.97, *p* = 0.039, adjusted *R*^2^ = 0.08.

In the neutral blocks, the number of failed trials had a significant effect on frustration ratings, *F*(1, 64) = 4.37, *p* = 0.041, suggesting that frustration increased with more failed trials. The effect of group was not significant, *F*(1, 64) = 1.09, *p* = 0.301. Importantly however, the interaction between failed trials and the group was trend level significant, *F*(1, 64) = 3.56, *p* = 0.064. The interaction term (see Table 11) suggested that among HC frustration increased more strongly with an increasing number of failed trials than among PWE.

In the provocation blocks, the number of self-caused failed trials had no significant effect on the frustration ratings after the blocks. The interaction between failed trials and the group was not significant either, suggesting that the relationship between failed trials and feelings of frustration did not differ between the groups under provocation conditions. However, the negative direction of the interaction term may indicate a trend toward a weaker association between failures and frustration in the patient group compared to HC.

The trend-level interaction effect of the group and failed trials on frustration in neutral blocks is visualized in Figure 4D.

## Discussion

The present study aimed to assess aggressive and impulsive behavior in PWE compared to HC. For this purpose, self-report questionnaires and two experimental provocation paradigms (TAP, TPP) were used. The results revealed that PWE, especially those under therapy with Levetiracetam as the predominant group in our sample, reported increased levels of self-perceived aggression and impulsivity in the questionnaires. However, their overall behavior in the experimental tasks showed no elevated aggression compared to HC. In contrast, behavioral responses of patients tended to be less responsive to provocation, and they demonstrated decreased emotional reactivity compared to HC. Yet, when patients were emotionally affected, they showed higher aggression during the tasks, particularly in those reporting higher revengefulness and frustration. The results demonstrate complex associations between emotions and aggressive behavior that is influenced by many individual parameters and cannot simply be explained by patient status.

### Self-perceived aggression and impulsivity

In agreement with previous studies (Shehata and Bateh, 2009; Izci et al., 2016; Shakeshaft et al., 2021; Lee et al., 2022b) and consistent with the subjective trait aggression hypothesis, PWE reported higher levels of aggression and impulsivity than HC in the self-report questionnaires. Medium effect sizes were observed for self-reported trait aggression, physical aggression and hostility, as well as for impulsivity and especially non-planning traits. Overall, these results suggest that PWE perceived themselves as both more aggressive and impulsive than HC.

At the same time, however, patients also reported more depressive symptoms and had more psychiatric comorbidities. Results suggest an influence of a psychiatric diagnosis on self-perception of aggression and impulsivity, with elevated self-reports being connected to co-occurring psychiatric diseases in the epilepsy group.

Prior research showed that PWE have an increased risk for depression and mental health disorders (Tellez-Zenteno et al., 2007; Chu, 2022). The influence of these psychiatric comorbidities that we ascertained here is also supported by previous studies, where mental health disorders, especially depression, were found to influence the quality of life and even the response to pharmacological treatment in PWE (Boylan et al., 2004; Hitiris et al., 2007; Abe et al., 2020). Depression is associated with negative cognitive biases, which can cause a more negative interpretation and evaluation of one’s thoughts and behaviors (Everaert et al., 2012; Noworyta et al., 2021). Patients with more depressive symptoms may generally view themselves more negatively, and therefore rate themselves as more aggressive and impulsive in questionnaires.

Additionally, stigma can have an influence on self-perception. Due to the chronicity of the illness and the visibility of certain seizure types, PWE are confronted with stigmatization. Higher stigma perception among patients is not only linked to more depressive symptoms (Tombini et al., 2020b) and may therefore further contribute to a negative self-image, but it also directly acts as a contributing factor for heightened self-reports of aggression (Seo et al., 2015).

### Affective and behavioral responses to provocation

Overall, the results revealed a clear divergence between trait measures, acute emotional states, and observed behavior in PWE. Although patients reported higher aggression, impulsivity, and depressive symptoms in the questionnaires, this pattern was not reflected in their emotional reactivity or their behavioral responses in the tasks. In both the TAP and the TPP, PWE showed weaker or absent changes in frustration and revengefulness following provocation, whereas HC demonstrated the expected increases in these state measures related to provocation. Likewise, while HC adapted their behavior in accordance with provocation in the TPP, PWE showed no corresponding behavioral adjustment.

Despite elevated trait reports, patients did not behave more aggressively than HC in either paradigm, supporting the subjective versus objective aggression hypothesis and indicating a dissociation between how patients perceive themselves and how they behave in controlled settings.

Several factors may explain this divergence between trait and state measures and observed behavior. The reduced emotional reactivity—visible in the lack of increased frustration and revengefulness—may reflect emotional blunting or reduced affect display. One reason may be problems in emotion regulation. PWE in the current sample indicated higher concealment and lower emotional tolerance in the ASQ compared to HC. Patients who rather suppress their acute emotions than accepting them may experience increased internal tension in the aftermath while not reporting increased negative states directly.

Adolescents with epilepsy showed reduced brain activity in areas related to error evaluation and learning and decision making (King et al., 2024), which may underlie the weakened emotional reactions to both induced and self-caused errors observed in the present study. Moreover, depressive symptoms, the perception of stigma, and impaired quality of life are factors that contribute to emotional dysregulation in PWE (Tombini et al., 2020a) and are associated with more negative self-perception.

Emotional suppression and concealment might also provide an explanation for the elevated trait reports of aggression. A previous study found that people who report greater tendencies to suppress emotions were more likely to show a projection bias, resulting in remembering past events and emotions more negatively than actually perceived at that time (Chang et al., 2018). Individuals with this biased memory may estimate their own aggressiveness as more intense and negative than this was the case. The same study also reported a connection between this projection bias and reductions in wellbeing over time. Although this aspect was not specifically investigated in this study, higher depressive symptoms may support the association between emotional suppression and reduced wellbeing.

Together, these factors suggest that the discrepancy between trait aggression, emotional states, and behavior in the TAP and TPP may result from a combination of emotional blunting, emotion regulation strategies, and biased self-perception. Further studies are needed to clarify the interaction of these mechanisms.

### Revengefulness and frustration as emotional predictors of behavior

One key finding from this study concerns the motivation for aggression—especially revengefulness in social contexts like the TAP and frustration in non-social contexts like the TPP- and its influence on observed aggressive behavior. Although the social and non-social provocation hypotheses were not supported, the data indicated that under specific conditions, emotional states can drive aggressive behavior in PWE.

During the high provocation block in the TAP, higher revengefulness was associated with increased money deduction in the patient group. Thus, while PWE overall did not exhibit more aggressive behavior or a stronger desire for revenge, those who reported high levels of revengefulness were more likely to act aggressively. This suggests that the expression of aggressive behavior in the patient group might be strongly influenced by their motivation, and only emergent under strong provocation conditions. This pattern was not observed in the HC group.

Krämer et al. (2007) found that the decision to retaliate, even before an actual aggressive act, activates the dorsal striatum that is also involved in reward processing. Striatal activity was observed when participants made the decision to punish their opponent, suggesting that the thought of revenge itself is a rewarding feeling. While PWE in the current study appeared to give in to the rewarding desire for revenge by deducting more money, HC did not increase their money deductions. Previous it was shown that healthy participants under provocation not only showed activation in striatal areas, but also displayed activation in regulatory regions, related to reduced retaliatory aggression (Krämer et al., 2007; Chester and DeWall, 2016).

### Gender differences

In the TAP, male participants behaved more aggressively than female participants, consistent with previous findings in clinical and non-clinical samples (Zeichner et al., 2003; Archer, 2004; Björkqvist, 2018).

Notably, in the TPP, male participants showed lower average AUCs than female participants. This result is contrary to the behavior demonstrated in the TAP and to the general assumption that males are more engaged in aggression.

In a study by Mathew et al. (2020) males and females performed a visuo-manual tracking task. Men showed better accuracy in being able to track a target with their hand compared to women, particularly through faster visuomotor decision processes (Mathew et al., 2020). One possible explanation may be that higher AUCs in women are a sign of less accurate and slower joystick use, and not necessarily depict a more aggressive behavior.

Another approach to understanding the contrary results from the two tasks is looking at the different provocation mechanisms. Previous research has shown that females and males tend to engage in different types of aggression. Women often favor more indirect forms of aggression, such as rumor spreading and social exclusion, while men tend to display physical, overt aggression (Vaillancourt, 2013; Denson et al., 2018). A meta-analysis conducted by Bettencourt and Miller (1996) showed that provocation has a moderating effect on gender aggression differences. While males behave more aggressive under unprovoked conditions, this difference often diminishes when provocation is applied (Bettencourt and Miller, 1996). In our study, women might have been more susceptible to the applied provocation in the TPP and as a result showed a stronger overall joystick use.

The unequal gender distribution between the PWE and HC group represents a potential confounding factor in this study, as gender differences in aggression, reactivity and task performance may influence self-reports and behavioral outcomes. Aggression might be overestimated in the PWE group, as this group has a higher proportion of male participants than the HC group, for whom physical and direct aggression is reported as higher than in females (Archer, 2004).

### Clinical implications

The findings of this study have several clinical implications for the understanding and management of PWE. First, our study highlights the complexity of aggression-related behavioral problems in epilepsy. Responses to provocation are likely shaped by multiple factors. While we cannot provide the main reason for increased aggression in this manuscript and it may be an effect of a certain type of ASM or epilepsy, we want to emphasize the need for further research that considers both the specific context in which aggression occurs as well as broader aspects of emotion regulation. Second, the discrepancy between elevated self-perceived aggression and impulsivity and the lack of increased aggressive behavior in experimental settings challenges some previous assumptions regarding aggressive behavior in PWE. It highlights the importance of a distinctive understanding of emotional and behavioral functioning in this patient group. Overreliance on self-reported measures should be avoided. Self-reported measures might rather reflect internal states and feelings, influenced by psychiatric comorbidities, than observable behavioral consequences.

The influence of psychiatric comorbidities, especially on self-reports, underlines the value of mental health screening and treatment in epilepsy care. Assessments for depressive symptoms, trauma disorders and emotion regulation should be implemented in the routine workup of PWE. Patients in this study showed signs of decreased reactivity and increased emotional blunting and suppression. Clinical attention should not only be given to emotional intensity, but also to how patients manage and express their emotions. Psychological support and therapy may improve quality of life and help develop more accurate self-perceptions.

Regarding adverse drug reactions of ASM, the absence of observable increased aggression suggests that emotional reactions should be monitored closely and put into relation with behavior. Clinicians should try to differentiate between perceived and actual behavioral changes, avoiding unnecessary medication adjustments.

### Limitations

Though the sample allowed overall comparisons between patients on ASM and HC, the limited sample size in the patient group was not sufficient for subgroup analyses regarding individual drugs. An overrepresentation of patients treated with Levetiracetam may also limit the generalizability over all investigated drugs.

Additionally, epilepsy is a disorder with many different etiologies that could involve a variety of repercussions on patient behavior. Due to the sample size, sub-analyses on these patient characteristics were not feasible.

Despite using validated and well-established experimental paradigms, both in a social and non-social context, these scenarios may not reflect real world experiences and aggressive behavior, especially as they have been conducted in an MRI setting, limiting movement and natural reactions.

## Conclusion

This study combined self-reports and experimental approaches to examine aggression in patients with epilepsy. Although patients reported higher levels of aggression in questionnaires compared to healthy controls, their behavioral responses in experimental provocation tasks did not reveal increased aggression. Patients showed reduced emotional reactivity, and aggression emerged especially when feelings of revengefulness or frustration were high. Our findings suggest a dissociation between perceived and observable aggression in patients with epilepsy and emphasize the importance of considering emotion regulation and psychiatric comorbidities when evaluating reports of behavioral adverse drug reactions of ASM, noting that the present conclusions primarily reflect patients treated with Levetiracetam.

## Data Availability

The datasets presented in this study can be found in online repositories. The names of the repository/repositories and accession number(s) can be found at: https://osf.io/pfb2c/overview.
