# Supplementary material for: Examining aggressive behavior in patients with epilepsy under treatment with Levetiracetam, Brivaracetam and Perampanel: a comparison to healthy controls
Source: Front Behav Neurosci. 2026 Apr 13;20:1799427. doi: 10.3389/fnbeh.2026.1799427 (PMC13111316; doi:10.3389/fnbeh.2026.1799427)
Supplement: Supplementary file 1 [file Data_Sheet_1.docx]

**Supplementary data**

**Inclusion of a SCID diagnosis in the TAP model:**

modelATEP8new <- lmer(Money~ HP_Group +TAP_condition + TAP_Version + Gender + TAP_condition:TAP_Revengefulness:HP_Group + SCID_diagnosis + (1|Study_ID), na.action ="na.omit", data = ATEP)

**Supplementary Table 1.** Statistical measures of the linear mixed model for aggression levels in the TAP with SCID diagnosis as covariate

|  | **df num** | **df den** | ***F*** | | ***p*** |
| --- | --- | --- | --- | --- | --- |
| Group (PWE / HC) | 1 | 116.98 | 0.99 | .323 | |
| SCID diagnosis | 1 | 61.89 | 1.31 | .257 | |
| TAP condition | 2 | 132.09 | 4.32 | .015 | |
| TAP Version | 1 | 61.91 | 0.91 | .344 | |
| Gender | 1 | 62.21 | 4.12 | .047 | |
| Study group x TPP condition x Revengefulness | 6 | 140.01 | 2.06 | .062 | |

**Inclusion of a SCID diagnosis in the TPP model:**

modelATEP_TPP_AUC5_new <- lmer(AUC_block~ HP_Group + TPP_block + SCID_diagnosis + Gender + TPP_Frustration:TPP_block:HP_Group + (1|SNumber), na.action ="na.omit", data = ATEP_TPP)

**Supplementary Table 2.** Statistical measures of the linear mixed model for the joystick area under the curve in the TPP with SCID diagnosis as covariate

|  | **df num** | **df den** | ***F*** | ***p*** |
| --- | --- | --- | --- | --- |
| Group (PWE / HC) | 1 | 124.22 | 0.44 | .501 |
| SCID diagnosis | 1 | 64.73 | 0.00 | .983 |
| TPP condition | 1 | 64.76 | 1.55 | .217 |
| Gender | 1 | 64.65 | 6.38 | .014 |
| Study group x TPP condition x Frustration | 4 | 89.31 | 3.19 | .017 |
